# Sertoliform cystadenoma: a rare benign tumour of the rete testis

**DOI:** 10.1186/1746-1596-8-23

**Published:** 2013-02-14

**Authors:** Felix Bremmer, Stefan Schweyer, Carl Ludwig Behnes, Manfred Blech, Heinz Joachim Radzun

**Affiliations:** 1 Department of Pathology, University of Göttingen, Göttingen, Germany; 2 Department of Urology, Helios Albert Schweitzer Hospital, Northeim, Germany; 3 Gemeinschaftspraxis Pathologie, Starnberg, Germany

## Abstract

**Abstract:**

Sertoliform cystadenoma of the rete testis represents an uncommon benign tumour. They appear in patients from 26 to 62 years of age. We describe a case of a 66-year-old man with a tumour in the area of the epididymal head. The tumour markers were not increased. Under the assumption of a malignant testicular tumour an inguinal orchiectomy was performed. The cut surface of this tumour was of grey/white color and showed small cysts. The tumour consisted of two compartments. The epithelial like tumour cells showed a sertoliform growth pattern and cystic dilatations. In between the tumour cells repeatedly actin expressing sclerotic areas could be recognized as the second tumour component. Proliferative activity was not increased. Immunohistochemically the tumour cells were positiv for inhibin, S-100, and CD 99. Alpha feto protein (AFP), human chorionic gonadotropin (ß-HCG) and placental alkaline phosphatase (PLAP) as well as synaptophysin, epithelial membrane antigene (EMA), and BCL-2 were not expressed. As far as we know this is the sixth reported case of this tumour. Because of the benign nature of this tumour the correct diagnosis is important for the intra- and postoperative management. Here we present a case of this rare tumour and discuss potential differential diagnosis.

**Virtual Slides:**

The virtual slide(s) for this article can be found here: http://www.diagnosticpathology.diagnomx.eu/vs/1956026143857335

## Clinical features

**Figure 1:**
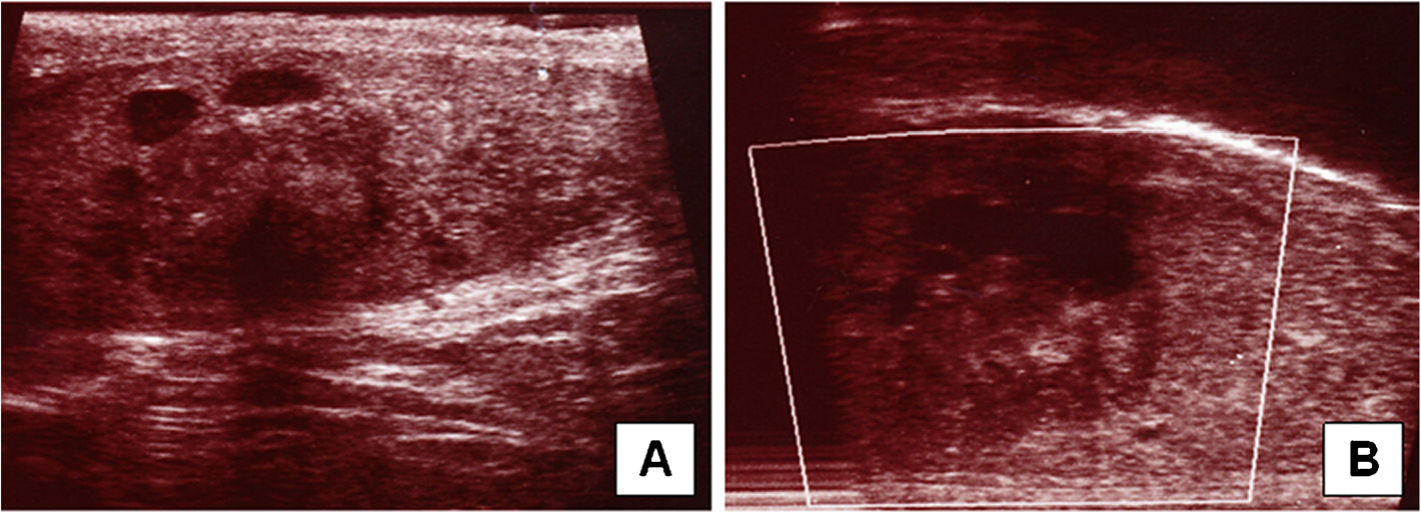
**Sertoliform cystadenoma of the rete testis:** On ultrasound examination the tumour arises from the rete testis (white arrow) and shows solid and cystic areas (black arrows, **A + B**).

A 66 year old man was presented in the urological clinic because of slightly increased blood level of prostatic specific antigen. The investigation of the prostate revealed a benign hyperplasia. Further urological examinations showed a mass of approximately 2 cm in diameter in the area of the epididymal head (Figure 1A+B). On ultrasound examination this mass proved to be cystic and irregularly bounded. The tumour markers alpha-fetoprotein (AFP) and human chorionic gonadotropin (ß-HCG) were not increased. Under the assumption of a malignant testicular tumour an inguinal orchiectomy was performed.

## Pathological findings

### Macroscopy

On macroscopical examination the testis measured 8.5 × 4.5 × 1.5 cm. The epidymidis measured 5 × 2 × 1.5 cm and the spermatic cord was 10 cm in length. The cutting surface of the testis showed a homogenous brown colour. Within the area of the tunica albuginea/rete testis a 2.1 × 1.8 × 1,4 cm tumour was detectable. The cut surface of this tumour was of grey/white color and showed small cysts of about 5 mm in diameter. The tumour exhibited an expansive growth pattern into the testis parenchym.

## Histology

**Figure 2:**
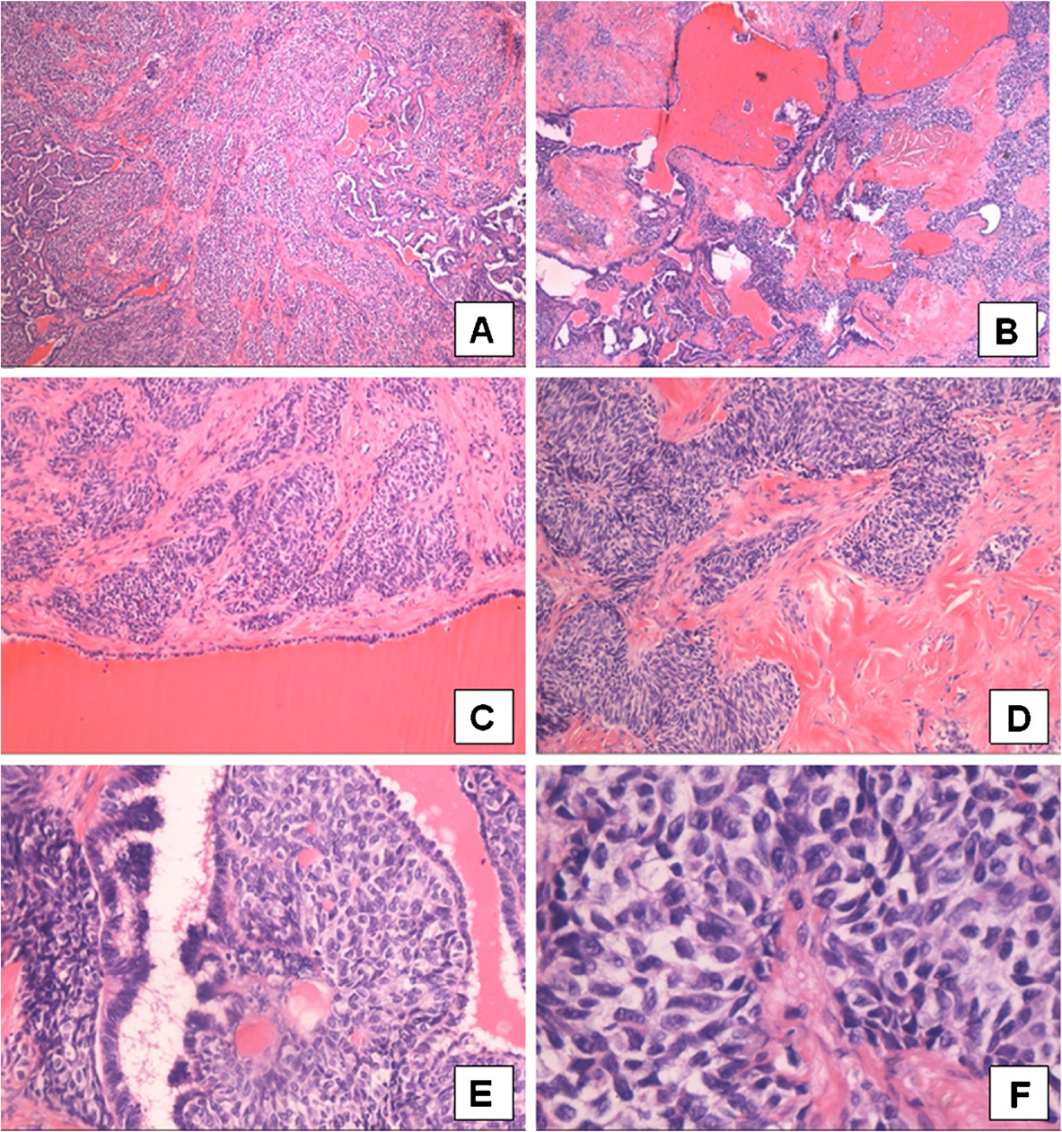
**Sertoliform cystadenoma of the rete testis:** The tumour shows solid (**A, H & E**, ×40) and cystic areas (**B, H & E**, ×40). Between the tumour cells and cystic structures sclerotic aereas can be seen (**C** + **D**, **H** &**E**, ×200). The tumour arises from the rete testis (**E**, arrow, **H&E**, ×400) and shows a sertoliform growth pattern (**F**, **H&E**, ×400).

**Figure 3:**
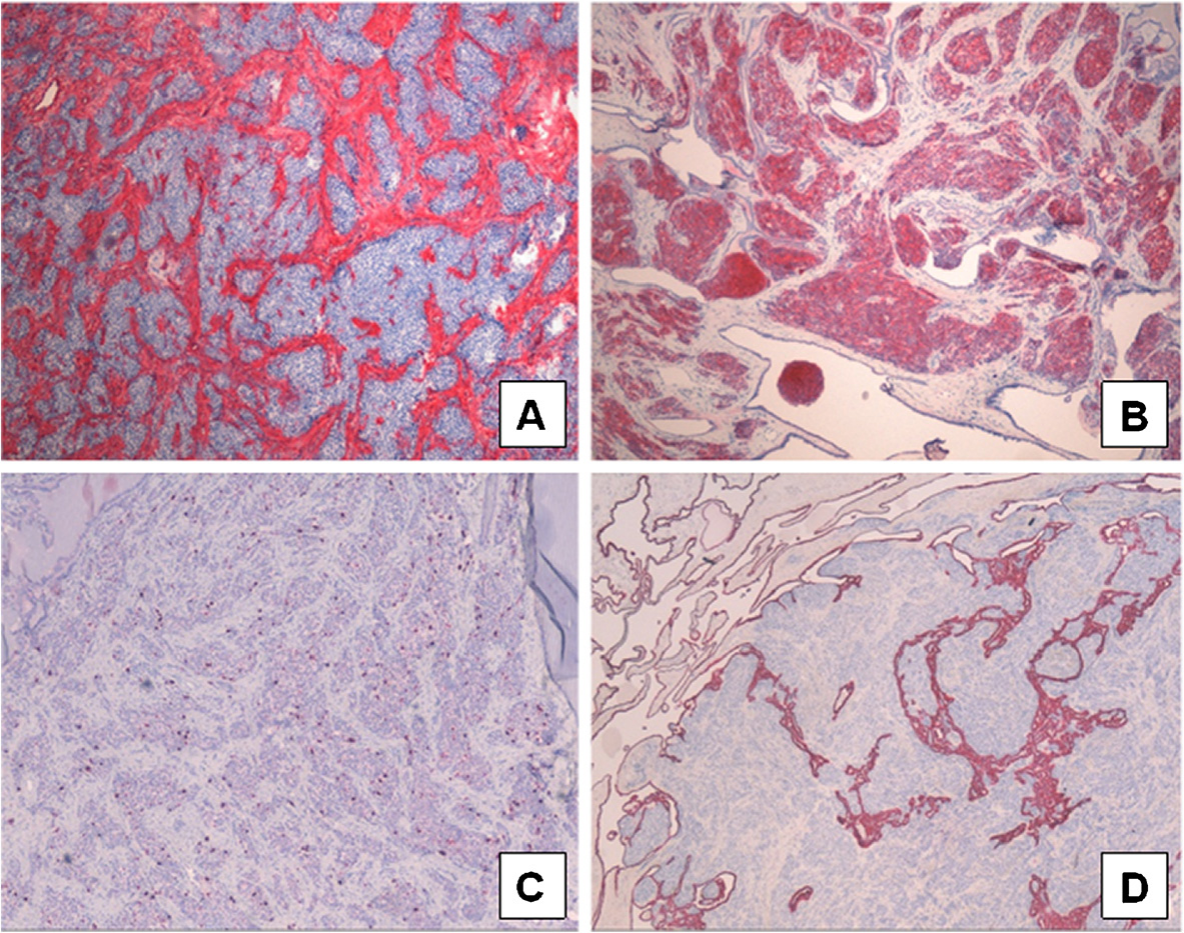
**Immunohistochemical analysis:** Sclerotic areas express actin (**A**; x40); Epithelial like tumour cells express inhibin (**B**; ×40). The Ki67 staining shows a low proliferative activity (**C**; ×40); Keratin expression revealed the rete testis penetrated by the tumour cells (**D**; ×40).

The tumourfree testicular tissue showed regular tubules, regular spermatogenesis, and normal interstitial tissue. The tumour consisted of two compartments. The epithelial like tumour cells showed a sertoliform growth pattern and cystic dilatations. The uniform tumour cells were ordered in tubules and acini. The cytoplasm of the tumour cells was eosinophilic, the nuclei showed prominent nucleoli (Figure 2A-F). Proliferative activity revealed by Ki-67 staining was not increased (Figure 3C). In between the tumour cells repeatedly actin expressing sclerotic areas could be recognized as the second tumour component (Figure 3A). Immunohistochemical examination of the epithelial like tumour cells revealed positivity for inhibin (Figure 3B), S-100, and CD 99. The germ cell markers such as AFP, ß-HCG and placental alkaline phosphatase (PLAP) as well as synaptophysin, epithelial membrane antigene (EMA), and BCL-2 were not expressed. Keratin expression could not be seen in both tumour elements but revealed the cystic alterated rete testis invaded by the tumour (Figure 3D). Because of the sertoliform growth pattern, the cystic areas and the origin of the tumour from the testis a sertoliform cystadenoma of the rete testis was diagnosed. This diagnosis was attested by special opinion.

## Discussion

So far only five cases of a sertoliform cystadenoma of the rete testis have been reported. They appear in patients from 26 to 62 years of age. The tumors range from 1 to 3 cm in size showing cystic and solid masses arising from the rete testis [1-3]. Sinclair et al. described a strong expression of inhibin and calretinin, and a focal expression for MF116, S-110, and CD99. Chromogranin, synaptophysin, CD56, PLAP, EMA, carcinoembryonic antigen (CEA) and CD15 were not expressed [2].

Because of the benign nature of sertoliform cystadenomas the correct diagnosis is important for the intra- and postoperative management. Clinically and on ultrasound examination a clear diagnosis can not be ultimatively made.

Because of the striking sclerotic tumour component three possible differential diagnoses exist morphologically: (1) Rete testis cystadenoma: This rare tumour also shows cystic dilatations and sclerotic areas within the rete testis, in addition the cysts are also lined by cuboidal epithelium. In contrast to sertoliform cystadenoma, however a sertoliform growth pattern is missing [1]. (2) Testicular sertoli cell tumour: Nearly the same tumour cell components with identical immunhistochemical staining can be seen. Because of its location inside the testis and lacking of cystic formations a clear differentiation from sertoliform cystadenoma can be made [4]. (3) Desmoplastic small round cell tumour: This tumour consists of uniform small cells with round nuclei and prominent cell borders supported by a prominent desmoplastic stroma. The tumour cells express desmin, neuron specific enolase (NSE), EMA, and vimentin and show a high proliferative activity [5].

## Conclusions

A sertoliform cystadenoma of the rete tesis is an extremly rare tumour of the testis. As far as we know this is the sixth reported case of this tumour. Because of the benign nature of this tumour the correct diagnosis is important for the intra- and postoperative management.

## Consent

Written informed consent was obtained from the patient for publication of this case report and any accompanying images. A copy of the written consent is available for review by the Editor-in-Chief of this journal.

## Competing interests

The authors declare that they have no competing interests.

## Authors’ contributions

FB and CLB drafted the manuscript and arranged histological pictures. FB, SS and HJR were responsible for microscopic and histopathologic elements. MB cared for the patient and provided clinical information. HJR was responsible for the critical revision of the manuscript. All authors read and approved the final manuscript.
